# Preventable hospitalization and access to primary health care in an area of Southern Italy

**DOI:** 10.1186/1472-6963-7-134

**Published:** 2007-08-30

**Authors:** Paolo Rizza, Aida Bianco, Maria Pavia, Italo F Angelillo

**Affiliations:** 1 Chair of Hygiene, Medical School, University of Catanzaro "Magna Græcia", Catanzaro, Italy; 2 Department of Public, Clinical and Preventive Medicine, Second University of Naples, Naples, Italy

## Abstract

**Background:**

Ambulatory care-sensitive conditions (ACSC), such as hypertension, diabetes, chronic heart failure, chronic obstructive pulmonary disease and asthma, are conditions that can be managed with timely and effective outpatient care reducing the need of hospitalization. Avoidable hospitalizations for ACSC have been used to assess access, quality and performance of the primary care delivery system. The aims of this study were to quantify the proportion of avoidable hospital admissions for ACSCs, to identify the related patient's socio-demographic profile and health conditions, to assess the relationship between the primary care access characteristics and preventable hospitalizations, and the usefulness of avoidable hospitalizations for ACSCs to monitor the effectiveness of primary health care.

**Methods:**

A random sample of 520 medical records of patients admitted to medical wards (Cardiology, Internal Medicine, Pneumology, Geriatrics) of a non-teaching acute care 717-bed hospital located in Catanzaro (Italy) were reviewed.

**Results:**

A total of 31.5% of the hospitalizations in the sample were judged to be preventable. Of these, 40% were for congestive heart failure, 23.2% for chronic obstructive pulmonary disease, 13.5% for angina without procedure, 8.4% for hypertension, and 7.1% for bacterial pneumonia. Preventable hospitalizations were significantly associated to age and sex since they were higher in older patients and in males. The proportion of patients who had a preventable hospitalization significantly increased with regard to the number of hospital admissions in the previous year and to the number of patients for each primary care physician (PCP), with lower number of PCP accesses and PCP medical visits in the previous year, with less satisfaction about PCP health services, and, finally, with worse self-reported health status and shorter length of hospital stay.

**Conclusion:**

The findings from this study add to the evidence and the urgency of developing and implementing effective interventions to improve delivery of health care at the community level and provided support to the usefulness of avoidable hospitalizations for ACSCs to monitor this process.

## Background

Ambulatory care-sensitive conditions (ACSC), such as hypertension, diabetes, chronic heart failure, chronic obstructive pulmonary disease and asthma, are conditions that can be managed with timely and effective outpatient care reducing the need of hospitalization. Rates of avoidable hospitalizations, also called preventable hospitalizations, for ACSC have been used to assess access, quality and performance of the primary care delivery system. This includes primary prevention, early detection and monitoring of acute episodes and follow-up and monitoring of chronic conditions.

The Agency for Healthcare Research and Quality (AHRQ) developed the Preventive Quality Indicators (PQIs), a set of measures to identify ACSC as rates of admission to the hospital based on the assumption that high hospitalization rates for ACSC may result from poor access to primary care and can be prevented [1]. ACSC have been evaluated in many countries most in the US but also in Australia, Canada and European countries [2-5] for determining the socioeconomic and medical conditions associated with hospitalized ACSC [6-12]; however, little is known about role of access to primary care, measured as number of accesses, number of patients per primary care physician (PCP), health care seeking and physician practice style [13-17].

In Italy, where universal and free access to primary health care is provided through the National Health Service, these indicators can serve as a convenient and effective evaluation tool to assess effectiveness of and barriers to access to primary care. Community health care is provided by health districts, geographical units responsible for coordinating and providing primary care, pharmaceuticals, home care, specialist and residential and semi-residential care. Primary care is provided by physicians and pediatricians working under government contract, who are paid a capitation fee based on the number of patients (adults or children) on their list with a maximum number of patients allowed (1500–1800 for general practitioners and 800–1000 for pediatricians). The PCPs act as "gate-keepers" for access to secondary services and this role has a great impact both on quality of outcomes and cost of healthcare, but for several reasons patients prefer to attend specialists or hospitals [18].

The aims of this study were to quantify the proportion of avoidable hospital admissions for ACSCs, to identify the related patient's socio-demographic profile and health conditions, to assess whether barriers to access to primary care were related to preventable hospitalizations in Italy and to assess the usefulness of avoidable hospitalizations for ACSCs to monitor the effectiveness of primary health care.

## Methods

### Study population

This cross-sectional study was conducted from April to July 2005 by reviewing a random sample of 520 medical records of patients aged 18 and over admitted to medical wards (Cardiology, Internal Medicine, Pneumology, Geriatrics) of a non-teaching acute care 717-bed hospital located in Catanzaro (Italy).

Two physicians, who were not involved in care and who had been previously trained, collected the data by reviewing charts and by interviewing at bedside all the patients who agreed to participate, independently. Actual data collection and extraction did not start until the performance of the reviewers had been judged satisfactory and showed satisfactory inter-rater reliability. The analysis on medical records was based on data reviewed by both physicians in which discrepancies were solved through discussion among reviewers.

A total of 492 patients agreed to participate and were enrolled for a response rate of 94.6%, thus minimizing the role of non-respondent bias.

### Review instrument

The following data were collected for each patient by reviewing charts: socio-demographics (age, gender, marital status, education level), distance of patient's home from hospital, Charlson et al. comorbidity index [19], ward and type of admission, and who referred the patient to the hospital. The following data were collected by interviewing at bedside all the patients who agreed to participate: socio-demographics (working activity, number of persons in the household), the self-reported health status, self-reported utilization of health services during the previous year (propensity to seek care, number and main reasons of visit and for not having been to a PCP medical visit, difficulty to the access to and satisfaction with PCP health services, number and main reasons for specialist visit, emergency access, hospital admission), and the name of their own PCP. The questionnaire focused particularly on frequency of utilization of health services during the previous year with most questions in "yes/no" format. If the answer was "yes" then the participants were asked the number of accesses. The questions on satisfaction and difficulty with PCP health services were scored on a four-point Likert scale with options for no, few, rather, and much. The questionnaire was pretested on a sample to ensure clarity of interpretation to improve the validity of responses. The accuracy of patient self-report of health services utilization is of paramount concern, however such data is often used to estimate health care utilization and have demonstrated to be a reasonably accurate method to obtain information on most types of medical utilization in the general population [20-22].

We selected 9 out of 16 PQIs from AHRQ, defined by diagnosis and procedure codes of International Classification of Diseases (ICD-9-CM) [23], to identify the preventable hospitalizations for ACS conditions: diabetes short-term and long-term complication, uncontrolled diabetes, chronic obstructive pulmonary disease, hypertension, congestive heart failure, bacterial pneumonia, angina without procedure and adult asthma. We excluded pediatric indicators (pediatric asthma, pediatric gastroenteritis, low birth weight) and indicators for adult conditions that were rarely encountered, in our setting, as discharge diagnosis, as measured by reviewing discharge records in the previous two years (perforated appendix, dehydration, urinary tract infection, lower-extremity amputation among patients with diabetes). Assessment of avoidable hospitalizations was made by using the discharge data according to the Guide to Prevention Quality Indicators from AHRQ [1].

The Ethics Committee of the "Mater Domini" Hospital of Catanzaro (Italy) approved the protocol of the study (Prot. E.C. n°16/2005).

### Statistical analysis

A multivariate logistic regression analysis was performed to identify baseline characteristics independently associated with the following outcomes of interest: preventable hospitalization for all investigated conditions, preventable hospitalization for cardiovascular diseases (hypertension, congestive heart failure, angina without procedure), preventable hospitalization for respiratory diseases (chronic obstructive pulmonary disease, bacterial pneumonia, adult asthma), preventable hospitalization for diabetes (diabetes short-term complication, diabetes long-term complication, uncontrolled diabetes). Model building strategy and particularly ways to include independent variables in the model (continuous, ordinal or categorical) took into account how each of these ways better fitted the data at the univariate analysis and we chose that way in the multivariate analysis. Moreover, as regards to the cut-off points for less or more than 12 accesses or visits per year, our idea was that in many chronic patients, one access per month is generally useful for monitoring chronic conditions. In all models the explanatory variables included were the following: patient's age (continuous), patient's sex (male = 0, female = 1), distance in kilometers between patient's home and hospital (continuous), educational level (no formal education = 0, primary school = 1, secondary school or higher = 2), living condition (with family = 1, other = 2), additional persons in the household (none = 0, 1 = 1, > 1 = 2), working activity (retired = 0, other = 1), type of admission (emergency physician = 1, other = 2), length of hospital stay (continuous), age-adjusted Charlson et al. comorbidity index (continuous), self-reported health status on a 10 points scale (≤ 4 = 0, ≥ 5 = 1), number of PCP accesses in the previous year (≤ 12 = 1, > 12 = 2), number of PCP medical visits in the previous year (≤ 12 = 1, > 12 = 2), satisfaction with PCP health services (no/few = 0, rather/much = 1), number of patients for each PCP (< 1000 = 1, 1000–1300 = 2, > 1300 = 3), number of specialist visits in community health services (none = 0, ≥ 1 = 1), number of emergency accesses in the previous year (none = 0, ≥ 1 = 1), and number of hospital admissions in the previous year (none = 0, ≥ 1 = 1). The significance level for variables entering the logistic regression models was set at 0.2 and for removing from the model at 0.4. Adjusted odds ratio (ORs) and 95% confidence intervals (CIs) were calculated. The data were analyzed using the Stata software program [24].

## Results

The main characteristics of the study population are presented in Table 1. Fifty-two per cent were females, the median age was 75 years (range 23–95), and three quarters lived with their family, more than half were in general medical wards (68.3%), the median length of stay was 9 days (range 1–83) and the median of Charlson et al. comorbidity index was 4 (range 0–14). Almost all (99.2%) had at least one PCP access in the previous year, more than 40% had at least one hospital admission in the previous year, and at least one district health services access. More than 70% were satisfied with PCP health services, and the main reasons for dissatisfaction were long waiting times for access (18.2%), opening hours (11.7%), and trust in hospital physicians (7.4%). Overall, 17.2% reported difficulty of access to PCP health services.

**Table 1:** Selected characteristics of the study population

Characteristic	N*	%
Sex		
Male	236	48
Female	256	52
Age group, years		
< 65	106	21.5
65–74	139	28.3
75–84	196	39.8
≥ 85	51	10.4
Median	75	
Education level		
No formal education	177	36
Primary school	186	37.8
Secondary school or higher	129	26.2
Marital status		
Married	286	58.1
Others	206	41.9
Living condition		
With family	368	74.8
Other	124	25.2
Additional persons in the household		
None	110	22.4
1	205	41.6
> 1	177	36
Working activity		
Retired	411	83.5
Other	81	16.5
Distance from home to hospital, km		
≤ 5	227	46.1
6–35	153	31.1
> 35	112	22.8
Median	20	
Type of admission		
Emergency physician	432	87.8
Other	60	12.2
Length of hospital stay, days		
Median	9	
Age-adjusted Charlson et al. comorbidity index		
Median	4	
Self-reported health status on a 10 points scale		
≤ 4	211	42.9
≥ 5	281	57.1
PCP accesses in the previous year		
None	4	0.8
1–12	129	26.2
> 12	359	73
PCP medical visits in the previous year		
≤ 12	304	62.3
> 12	184	37.7
Satisfaction with PCP health services		
No/few	135	27.7
Rather/much	353	72.3
Difficulty of access to PCP health services		
No/few	404	82.8
Rather/much	84	17.2
Patients for each PCP		
< 1000	146	29.9
1000–1300	137	28.1
> 1300	205	42
District health services accesses in the previous year		
None	310	63.5
≥ 1	178	36.5
Emergency accesses in the previous year		
None	260	52.8
≥ 1	232	47.2
Hospital admissions in the previous year		
None	275	55.9
≥ 1	217	44.1

*The numbers that do not add to 492 are due to not applicable data for the variable.

PCP, primary care physician.

In the study period, a total of 31.5% of the hospitalizations in the sample were judged to be preventable. Of these, 40% were for congestive heart failure, 23.2% for chronic obstructive pulmonary disease, 13.5% for angina without procedure, 8.4% for hypertension, 7.1% for bacterial pneumonia, 3.2% for diabetes short-term complication, 2.6% for adult asthma, 1.3% for diabetes long-term complication and 0.6% for uncontrolled diabetes.

Patients admitted for a preventable hospitalization were more frequently males (58.1% vs. 43.3%), older (median age 76 vs. 74), with a higher unsatisfactory self-reported health status (56.1% vs. 36.8%), reported more frequently less than 13 PCP medical visits (93.5% vs. 47.9%) and less than 13 PCP accesses (41.6% vs. 19.5%) in the previous year, attended a PCP with a higher number of patients (65.6% vs. 31.1%), were more frequently unsatisfied by PCP health services (54% vs. 15.6%), and were more likely to have had at least one emergency access (52.6% vs. 45.2%) and hospital admission in the previous year (54% vs. 40.1%).

Preventable hospitalizations were significantly associated with age and sex since they were higher in older patients (OR = 1.03, 95% CI = 1.01–1.05, p = 0.027) and in males (OR = 0.52, 95% CI = 0.31–0.87, p = 0.013). The proportion of patients who had a preventable hospitalization significantly increased with regard to the number of hospital admissions in the previous year (OR = 1.76, 95% CI = 1.06–2.93, p = 0.03) and to the number of patients for each PCP (OR = 2.25, 95% CI = 1.62–3.13, p < 0.001), with lower number of PCP accesses (OR = 0.52, 95% CI = 0.3–0.93, p = 0.027) and PCP medical visits in the previous year (OR = 0.1, 95% CI = 0.05–0.23, p < 0.001), with less satisfaction about PCP health services (OR = 0.34, 95% CI = 0.2–0.58, p < 0.001), and, finally, with worse self-reported health status (OR = 0.53, 95% CI = 0.31–0.89, p = 0.017) and shorter length of hospital stay (OR = 0.95, 95% CI = 0.91–0.99, p = 0.011) (Model 1 in Table 2).

**Table 2:** Logistic regression models results ^1,2^

Variable	OR	SE	95% CI	p
**Model 1. Outcome: Overall preventable hospitalization**				
**Log-likelihood = -194.19, χ**^2 ^**= 220.14, p < 0.001, Number of observations = 488**				
Number of PCP medical visits in the previous year	0.1	0.04	0.05–0.23	< 0.001
Number of patients for each PCP	2.25	0.38	1.62–3.13	< 0.001
Satisfaction with PCP health services	0.34	0.09	0.2–0.58	< 0.001
Length of hospital stay	0.95	0.02	0.91–0.99	0.011
Sex	0.52	0.14	0.31–0.87	0.013
Self-reported health status	0.53	0.14	0.31–0.89	0.017
Number of PCP accesses in the previous year	0.52	0.15	0.3–0.93	0.027
Age	1.03	0.01	1.01–1.05	0.027
Number of hospital admissions in the previous year	1.76	0.46	1.06–2.93	0.03
Additional persons in the household	0.75	0.13	0.53–1.06	0.105
Type of admission	0.57	0.24	0.25–1.3	0.18
Distance between patient's home and hospital	0.99	0.01	0.98–1.01	0.399
				
**Model 2. Outcome: Preventable hospitalization for cardiovascular diseases**				
**Log-likelihood = -150.13, χ**^2 ^**= 153.38, p < 0.001, Number of observations = 429**				
Number of patients for each PCP	2.2	0.43	1.5–3.22	< 0.001
Satisfaction with PCP health services	0.31	0.09	0.17–0.57	< 0.001
Number of PCP medical visits in the previous year	0.12	0.06	0.05–0.3	< 0.001
Length of hospital stay	0.94	0.02	0.9–0.99	0.013
Self-reported health status	0.5	0.16	0.27–0.93	0.027
Number of PCP accesses in the previous year	0.52	0.17	0.27–0.98	0.044
Working activity	0.37	0.18	0.13–0.97	0.044
Sex	0.62	0.18	0.35–1.1	0.106
Number of hospital admissions in the previous year	1.53	0.46	0.85–2.77	0.156
Additional persons in the household	0.76	0.15	0.51–1.13	0.172
Age-adjusted Charlson et al. comorbidity index	1.08	0.07	0.96–1.22	0.19
				
**Model 3. Outcome: Preventable hospitalization for respiratory diseases**				
**Log-likelihood = -92.36, χ**^2 ^**= 116.38, p < 0.001, Number of observations = 385**				
Number of patients for each PCP	2.86	0.8	1.66–4.94	< 0.001
Satisfaction with PCP health services	0.27	0.11	0.13–0.58	0.001
Number of PCP medical visits in the previous year	0.11	0.07	0.03–0.4	0.001
Sex	0.27	0.12	0.11–0.64	0.003
Number of emergency accesses in the previous year	2.71	1.12	1.2–6.11	0.016
Number of PCP accesses in the previous year	0.34	0.15	0.14–0.82	0.017
Type of admission	0.2	0.17	0.04–1.05	0.058
Living condition	2.35	1.08	0.95–5.81	0.063
Self-reported health status	0.49	0.21	0.21–1.12	0.092
Age	1.03	0.02	0.99–1.07	0.131
Age-adjusted Charlson et al. comorbidity index	0.88	0.08	0.73–1.05	0.159
Length of hospital stay	0.97	0.03	0.92–1.02	0.232
				
**Model 4. Outcome: Preventable hospitalization for diabetes**				
**Log-likelihood = -21.54, χ**^2 ^**= 18.77, p = 0.0277, Number of observations = 144**				
Number of patients for each PCP	4.75	3.29	1.22–18.48	0.025
Number of hospital admissions in the previous year	9.74	10.67	1.14–83.39	0.038
Number of PCP accesses in the previous year	0.15	0.17	0.02–1.34	0.090
Education level	0.27	0.23	0.05–1.46	0.129
Length of hospital stay	0.88	0.09	0.72–1.08	0.216
Number of specialist visits in community health services	0.36	0.37	0.05–2.79	0.326
Self-reported health status	2.61	2.57	0.38–18	0.332
Sex	2.47	2.33	0.39–15.65	0.338
Age	1.04	0.05	0.95–1.14	0.340

^1^Significance level is set at p ≤ 0.05

^2^The variables are presented in order of decreasing significance.

PCP, primary care physician.

Preventable hospitalizations for cardiovascular diseases were significantly more common for higher number of patients for each PCP (OR = 2.2, 95% CI = 1.5–3.22, p < 0.001) and for lower number of PCP accesses (OR = 0.52, 95% CI = 0.27–0.98, p = 0.044) and PCP medical visits (OR = 0.12, 95% CI = 0.05–0.3, p < 0.001) in the previous year. Moreover patients who had a preventable hospitalization for cardiovascular diseases were significantly more likely to be less satisfied for PCP health services (OR = 0.31, 95% CI = 0.17–0.57, p < 0.001), retired (OR = 0.37, 95% CI = 0.13–0.97, p = 0.044), with worse self-reported health status (OR = 0.5, 95% CI = 0.27–0.93, p = 0.027) and shorter length of hospital stay (OR = 0.94, 95% CI = 0.9–0.99, p = 0.013) (Model 2 in Table 2).

Preventable hospitalizations for respiratory diseases were significantly associated with higher number of patients for each PCP (OR = 2.86, 95% CI = 1.66–4.94, p < 0.001) and emergency accesses in the previous year (OR = 2.71, 95% CI = 1.2–6.11, p = 0.016), with males (OR = 0.27, 95% CI = 0.11–0.64, p = 0.003), worse satisfaction for PCP health services (OR = 0.27, 95% CI = 0.13–0.58, p = 0.001) and lower number of PCP accesses (OR = 0.34, 95% CI = 0.14–0.82, p = 0.017) and PCP medical visits (OR = 0.11, 95% CI = 0.03–0.4, p = 0.001) in the previous year (Model 3 in Table 2).

Finally, preventable hospitalizations for diabetes were significantly higher with the number of hospital admissions in the previous year (OR = 9.74, 95% CI = 1.14–83.39, p = 0.038) and the number of patients for each PCP (OR = 4.75, 95% CI = 1.22–18.48, p = 0.025) (Model 4 in Table 2).

## Discussion

One of the main findings of our study was that more than 30% of the hospitalizations were considered preventable according to the PQI indicators. Although most of the studies conducted on this topic used hospitalization rates [13,25-30], investigations on prevalence of avoidable hospitalizations showed variable results ranging from 5.9% in the USA considering 12 ACS conditions [31] to 34% in the same country [30]. Therefore, our prevalence is one of the highest encountered in the literature and deserves detailed comments. It has been argued that hospital admissions rates for ACSC alone are not sufficient proof that the provision of ambulatory care is inadequate, since some hospitalizations for ACSC will occur only because some ACSC are less manageable than others. Moreover, higher proportions of ACSCs may reflect higher prevalence for those particular diseases in the population. However, prevalence of the chronic diseases evaluated in our study is not substantially different from that encountered in most European countries, and we strongly believe that the high prevalence of admissions for ACSCs is related to unsatisfactory access and delivery of primary care, which leads to deterioration of health conditions and to overutilization of hospitals. Indeed, this conclusion has already been adduced in a survey conducted by some of us to assess satisfaction with PCPs which showed that many patients prefer to seek assistance at a more sophisticated level of care [18]. Moreover, we investigated emergency department use and hospitalization in the prior year as very interesting indicators of PCP access problems. Indeed, findings of a previous study conducted by some of us in the same area, regarding the utilization of the emergency department as a source of non-urgent care indicated high overutilization of the department and that patients were self-referred or referred by relatives to the emergency department [32]. This observation emphasizes that, although all patients in our national health care system have a general practitioner who should provide primary care, the emergency department is used as a primary health care facility and, therefore, this prevents general practitioners from playing an effective role in the patient management and as an interface between hospital and community based services. As a consequence, we agree that the health of patients with certain chronic medical conditions deteriorates without access to regular medical care and that this decline contributes to increased use of emergency departments and ultimately to increased hospitalizations [33,34]. Programs to improve the access and quality of primary care have notably reduced emergency department visits. Results of such programs suggest that areas with high levels of emergency department visits may have less access to primary care, and therefore poorer population health [35].

We found that avoidable hospitalizations are more likely to have a shorter length of stay. We have tested this association, since in previous studies performed in our area, high inappropriateness of hospital use was found and we believe that for chronic conditions in many cases this could happen also for avoidable hospitalizations and indeed inappropriate admissions have generally shorter length of stay [36].

Almost all prior studies have computed rates from databases, and factors not contained such as variations in disease prevalence, health care seeking behavior, and physician practice style could also affect preventable hospitalization rates [13], and it has been reported that the study of predictors of ACSC hospitalizations should have been applied to individual-level data [17]. We examined preventable hospitalizations in individual patients, comparing subjects admitted for ACSC conditions to those admitted for other reasons, and this strategy allowed us to identify the independent effects of various factors, pertaining to subjects' characteristics and primary care supply, on the probability of ACSC admissions.

Regarding subjects' demographic and social characteristics we found that older males were more likely to be hospitalized for ACSCs, whereas no other socio-demographic feature tested predicted ACSCs. Preventable hospitalizations have been associated with lower socioeconomic status, advanced age, poor health and higher education [37], since people with higher levels of education are likely to have greater awareness and knowledge of the local health care system and a better knowledge of disease processes, and may therefore seek medical treatment earlier. In our study education did not influence admissions for ACSCs, whereas, as in a previous study [38], perceived poor health status was indeed a significant predictor of hospitalization for ACSCs.

Primary care access measures, such as satisfaction with PCP health services and number of patients per PCP, as well as proxies of propensity to seek care, such as number of accesses and visits to PCPs in the previous year, were all related to hospitalization for ACSCs. Our results indicate that poor access to primary health care increases the likelihood of hospitalization for ACSCs, after controlling for most of the other factors that may affect hospital admission, such as socio-demographics and propensity to seek care. Some of the variables included in the model, such as number of PCP accesses and visits in the previous year, number of patients per PCP and satisfaction with PCP services, may be highly correlated; therefore we calculated some selected correlation coefficients and we found that, as expected, number of PCP accesses and PCP visits were significantly correlated (r = 0.46, p < 0.001) and satisfaction with PCP services was significantly correlated to number of PCP visits (r = 0.30, p < 0.001), whereas number of patients per PCP was inversely correlated to number of PCP visits (r = -0.17, p < 0.001) and satisfaction with PCP health services (r = -0.19, p < 0.001). To assess the role of each of these variables independently from others, we included all of them into the logistic regression model. Therefore, we believe that our results strongly support the validity of ACSCs as an indicator of quality of primary care and confirm the crucial role of PCPs in reducing unnecessary hospitalizations. Similar findings have been reported by Zhan et al., who showed that living in a primary care shortage area or in a higher supply of hospital beds area represented independent risk factor for a preventable hospitalization, even after controlling for other socio-demographic characteristics associated with health care use [37]. Moreover, adults identified as having a PCP were four times more likely to be discharged with a non-ACSC diagnosis compared to those without a PCP [17].

We collected data in one hospital and concern about generalizability of our results may arise. However, although very scarce data is available in Italy on ACSCs, it seems, as for many other health services indicators, that there are differences between Northern and Southern Italy. In a recent report from the Observatory of the Health of the Italian Regions, avoidable hospitalization rates were similar in Southern regions and consistently higher as compared to Northern regions [39]. For example, for adult asthma the preventable hospitalization rates in Southern Italy ranged from 0.27 discharges/1.000 inhabitants in Campania and Sicily to 0.45 discharges/1.000 inhabitants in Sardinia (Calabria region has a rate of 0.35 discharges/1.000 inhabitants). In the centre and in the north of Italy rates ranged from 0.13 discharges/1.000 inhabitants in Valle d'Aosta to 0.33 discharges/1.000 inhabitants in Bozen's autonomous administration, whereas the overall national rate is 0.24 discharges/1.000 inhabitants [39]. Therefore, although we cannot exclude that our results pertain only to our area, it is reasonable to suppose that an analogous context may be referred to the Southern part of our country. To have more insight into provision of primary care in other areas as measured by avoidable hospitalization, we strongly suggest replication of the study in other regions of the country.

Most Western countries are facing tremendous transformations in their healthcare systems, and one main element of change concerns the transition from an inpatient hospital-centered health care system to a multi level community-spread healthcare system that allows selection of health conditions that can be treated only in hospital. However, this transition is far from being completely implemented in Italy and our findings confirm that improvement of access to primary care may reduce avoidable hospitalizations. Therefore, our results have significant implications for health care policymakers, since ACSCs may be useful to monitor effectiveness and to reduce barriers to access to primary health care.

## Conclusion

In conclusion, the findings from this study add to the evidence and the urgency of developing and implementing effective interventions to improve delivery of health care at the community level and provided support to the usefulness of avoidable hospitalizations for ACSCs to monitor this process.

## Competing interests

The author(s) declare that they have no competing interests.

## Authors' contributions

PR and AB participated in the design of the study, collected the data, and contributed to the data analysis and interpretation. MP designed the study, the data analysis and interpretation, and wrote the article. IFA designed the study, the data analysis and interpretation, and gave final approval of the version to be published.

All authors read and approved the final manuscript.

## Pre-publication history

The pre-publication history for this paper can be accessed here:


